# Vitamin D Supplementation Improves Serological Parameters and Recovery Outcomes in COVID-19 Patients

**DOI:** 10.2174/0118715303457486260113044737

**Published:** 2026-03-11

**Authors:** Zhaoying Chen, Jianping Sheng, Qihang Ma, Wanpeng Wang, Lijie Wang, Lin Yang, Lin Liu

**Affiliations:** 1 Department of Endocrinology, Weifang People’s Hospital, Shandong Second Medical University, Weifang 261000, China;; 2 Shandong Second Medical University, Weifang 261053, China;; 3 Department of Metabolic Diseases and Weight Management, Weifang People’s Hospital, Shandong Second Medical University, Weifang 261000, China;; 4 Department of Infectious Diseases, Weifang People’s Hospital, Shandong Second Medical University, Weifang 261000, China

**Keywords:** Vitamin D, COVID-19, inflammation, therapeutic efficacy, clinical recovery, imaging findings

## Abstract

**Introduction:**

This study aimed to investigate the effects of vitamin D on serological, etiological, and imaging indicators in patients with Coronavirus Disease 2019 (COVID-19), exploring new strategies for its prevention and treatment.

**Methods:**

In this retrospective observational study, 300 COVID-19 patients admitted between January 2022 and March 2023 were enrolled from a public health center and our hospital. Participants were stratified into standard treatment (n=150) and vitamin D supplementation (n=150) groups. Outcomes included clinical recovery and improvements in laboratory, etiological, and imaging findings.

**Results and Discussion:**

No significant intergroup differences were observed at baseline in demographic or laboratory parameters, including white blood cell (WBC), neutrophil (NEUT), lymphocyte (LYM), interleukin (IL)-6, c-reactive protein (CRP), procalcitonin (PCT), and serum calcium (*P* > 0.05). Both groups exhibited subnormal 25(OH)D levels initially. After adjusting for age, sex, hypertension, diabetes, and cardiovascular and cerebrovascular diseases, multivariable linear regression analyses indicated that the vitamin D group showed significant increases in 25(OH)D and serum calcium, while the standard treatment group exhibited only a mild increase in 25(OH)D with no significant change in serum calcium after treatment. Both groups showed decreases in WBC, NEUT, IL-6, CRP, and PCT, and increases in LYM after treatment. Compared with the standard group, the vitamin D group had greater improvements in NEUT, LYM, 25(OH)D, and serum calcium (*P* < 0.05), and more pronounced reductions in IL-6 and CRP (*P* < 0.05). Symptom resolution and viral clearance times were significantly shorter in the vitamin D group (*P* < 0.05), and imaging improvements were more evident (*P* < 0.05).

**Conclusion:**

Supplementation with vitamin D, in addition to standard treatment, improves inflammatory markers, shortens the disease course, and is associated with radiographic improvement in COVID-19 patients.

## 1 INTRODUCTION

Corona virus disease 2019 (COVID-19) primarily manifests as respiratory symptoms and is highly heterogeneous [1]. Mild cases may present only with fever, fatigue, and dry cough, while severe cases can lead to acute respiratory distress syndrome (ARDS), septic shock, uncorrectable metabolic acidosis, and coagulation dysfunction [2]. In its early stages, COVID-19 is highly contagious and has an extremely high mortality rate, posing a threat to global health and urgently requiring public health measures to reduce infection risk, disease severity, and mortality [3, 4]. Even today, the global COVID-19 situation continues to exhibit fluctuating trends with resurgences in certain regions. Particularly for high-risk populations, the virus may act as a precipitating factor, exacerbating illness [5]. Sustained vigilance remains essential to alleviate the long-term impact of COVID-19.

Vitamin D is a steroid hormone that can be synthesized in the skin from 7-dehydrocholesterol upon UV exposure [6], or obtained exogenously from food and supplements. Besides its role in regulating calcium and phosphorus, vitamin D also has anti-infective, anti-inflammatory, and immune- modulating effects [7, 8]. Vitamin D deficiency is a public health problem affecting over one billion children and adults worldwide. Research shows that vitamin D deficiency is linked to various acute and chronic diseases, including infectious diseases, autoimmune diseases, type 2 diabetes, cancer, cardiovascular disease, and neurological disorders [9-12]. The elderly, smokers, obese individuals, patients with chronic diseases like diabetes and hypertension, and African Americans are more prone to vitamin D deficiency, and these populations overlap significantly with high-risk groups for poor COVID-19 outcomes [13]. A retrospective cross- sectional study in Wuhan, China, found that the mean serum vitamin D levels in COVID-19 patients were significantly lower than in the control group, and vitamin D levels were negatively correlated with disease severity [14]. This suggests that vitamin D deficiency may be a significant risk factor for COVID-19. Most studies from other countries show that vitamin D supplementation can reduce the risk of COVID-19 infection [15, 16], lessen disease severity [17, 18], slow disease progression [17], and lower the mortality rate [19]. However, this view is not universally agreed upon, as some studies suggest that vitamin D treatment provides no significant benefit in improving poor outcomes for COVID-19 [20, 21]. To date, there is no definitive conclusion on vitamin D intervention studies. This study collected and organized data from COVID-19 patients treated on the front lines of pandemic control, observing the effects of adding vitamin D to standard treatment on symptom resolution time, nucleic acid negative conversion time, and changes in etiological, serological, and imaging indicators. By comparing the changes in these indicators, we aimed to further verify the therapeutic effect of vitamin D on COVID-19 and explore the underlying mechanisms.

## 2 MATERIALS AND METHODS

### 2.1 Study Subjects

This was a retrospective observational study in which patients were categorized into the vitamin D group or the standard treatment group according to whether intramuscular vitamin D2 injection was administered as part of routine clinical care. We collected data from COVID-19 patients who sought medical attention at the city's public health center and Weifang People's Hospital from January 2022 to March 2023. After excluding cases with incomplete data and those who were lost to follow-up, 300 patients were included, comprising 174 males and 126 females. This study was approved by the Medical Ethics Committee of Weifang People's Hospital (KYLL20231101-14).

### 2.2 Inclusion and Exclusion Criteria

#### 2.2.1 Inclusion Criteria

COVID-19 patients were enrolled based on the diagnostic criteria outlined in the “Diagnosis and Treatment Protocol for Novel Coronavirus Infection (Trial Version 10)”. Eligible patients met the following criteria: (1) presence of clinical manifestations associated with severe acute respiratory syndrome coronavirus-2 (SARS-CoV-2) infection; and (2) a positive SARS-CoV-2 nucleic acid or antigen test result.

#### 2.2.2 Exclusion Criteria

(1) Severe COVID-19 patients who met the criteria for severe/critical clinical classification in the “Diagnosis and Treatment Protocol for Novel Coronavirus Infection (Trial Version 10)”, such as those with shortness of breath, dyspnea, significant progression on imaging, impaired consciousness, other organ failure, or ARDS.

(2) Those with contraindications to vitamin D supplementation, such as hypercalcemia, vitamin D intoxication, or hyperphosphatemia with renal rickets.

### 2.3 Research Methods

#### 2.3.1 Clinical Data

We collected personal information of the study subjects, including name, gender, age, and past medical history, and inquired in detail about the onset of the disease: time of onset, symptoms and signs, treatment process, and time to symptom resolution (the latest time when body temperature returned to normal, spirit and physical strength recovered, and symptoms like sore throat and cough disappeared; for cases with symptom recurrence, the time of the last symptom disappearance was used).

#### 2.3.2 Biochemical and Imaging Examinations

We recorded the initial positive and negative conversion times of the COVID-19 nucleic acid test (for cases that tested positive again after a negative result, the time of the last negative result was used) to calculate the number of days required for nucleic acid conversion. We also recorded pre-treatment and 5-day post-treatment levels of white blood cell (WBC), neutrophil (NEUT), lymphocyte (LYM), interleukin (IL)-6, c-reactive protein (CRP), procalcitonin (PCT), and serum calcium, as well as pre-treatment and 1-month post-treatment levels of 25(OH)D. Imaging platforms were used to query chest (lung) CT changes before treatment and 5 days after treatment. Based on the changes in the lesions, they were categorized as no change, improvement, or worsening.

#### 2.3.3 Treatment Protocol

Patients were divided into a vitamin D group and a standard treatment group based on whether vitamin D was added to their treatment. The treatment protocols for both groups were developed based on the consensus opinions of the city's public health center expert group. The standard treatment group received symptomatic treatment with antibiotics like cefoperazone-sulbactam and piperacillin-tazobactam for anti-infection, methylprednisolone for anti-inflammation, and oseltamivir for antiviral therapy. The vitamin D group received a single intramuscular injection of Vitamin D2 Injection (Jiangxi Gannan Haixin Pharmaceutical Co., Ltd.) 15mg, in addition to the standard treatment. Both groups were given calcium supplements based on their electrolyte levels. In this study, a single intramuscular injection of 15 mg vitamin D_2_ (equivalent to 600,000 IU) was administered. This dose was selected based on clinical guidelines and pharmacokinetic studies for the management of severe vitamin D deficiency. According to the Chinese Guidelines for the Diagnosis and Management of Vitamin D Deficiency and Osteoporosis (2020) and the Endocrine Society Clinical Practice Guidelines, a high-dose bolus regimen (typically 300,000–600,000 IU) is recommended for adults with severe deficiency (25(OH)D < 10 ng/mL) to rapidly raise serum concentrations to optimal levels. Furthermore, several clinical trials in COVID-19 patients have employed similar high- dose vitamin D supplementation (ranging from 100,000 to 600,000 IU in a single dose), demonstrating both safety and immunomodulatory benefits [22]. The 15 mg (600,000 IU) dose used in this study was chosen to ensure effective correction of vitamin D deficiency within a short timeframe while minimizing the risk of adverse effects such as hypercalcemia.

### 2.4 Statistical Methods

Data were analyzed using SPSS 27.0 statistical software. Quantitative data conforming to a normal distribution were expressed as mean ± standard deviation (Mean ± SD) and analyzed using an independent sample t-test; quantitative data not conforming to a normal distribution were expressed as median (interquartile range) [M(Q1, Q3)] and analyzed using a non-parametric test. Count data were expressed as percentages (%) and analyzed using the chi-square test. A *P* < 0.05 was considered statistically significant. For confounder-adjusted comparisons, multivariable linear regression analyses were performed with the change (difference) values of serological markers as dependent variables and group as the main independent variable, adjusting for age, sex, hypertension, diabetes, and cardiovascular and cerebrovascular diseases. AI tool disclosure: We occasionally consulted OpenAI ChatGPT (GPT-5.2) when preparing the confounder-adjusted analyses. All computations were performed by the authors using SPSS 27.0, with the final outputs and interpretations checked by the research team. The AI tool did not receive any raw participant-level or identifiable information. Additionally, a post hoc power analysis was conducted using G*Power software (version 3.1.9.7). Based on the observed effect sizes for the primary inflammatory markers (CRP and IL-6) and a sample size of 150 per group, the calculated statistical power (1−β) at a two-sided significance level of 0.05 was greater than 95%.

## 3 RESULTS

### 3.1 Comparison of Baseline Data between the Vitamin D Group and the Standard Treatment Group

This study included 300 COVID-19 patients, who were divided into a standard treatment group and a vitamin D group based on whether they received vitamin D supplementation. The standard treatment group had 150 patients, with 89 males and 61 females (59.3% male/40.7% female); the vitamin D group had 150 patients, with 85 males and 65 females (56.7% male/43.3% female). There were no differences in gender, age, or pre-treatment levels of WBC, NEUT, LYM, IL-6, CRP, PCT, 25(OH)D, or serum calcium between the two groups (*P* > 0.05). Moreover, the overall 25(OH)D levels in both groups were significantly below the normal lower limit (Table **1**).

### 3.2 Comparison of Serological Parameters before and after Treatment in the Vitamin D Group and the Standard Treatment Group

Compared with pre-treatment, the post-treatment levels of 25(OH)D and serum calcium were both elevated in the Vitamin D group (*P* < 0.05), whereas in the Standard Treatment Group, 25(OH)D only showed a slight increase compared with pre-treatment (*P* < 0.05), and serum calcium showed no significant difference (*P* > 0.05). Compared with pre-treatment, both groups showed decreased WBC and NEUT, and increased LYM levels after treatment (*P* < 0.05). Furthermore, compared with pre-treatment, post-treatment levels of IL-6, CRP, and PCT significantly decreased in both groups (*P* < 0.05) (Table **2**).

### 3.3 Comparison of Inter-group Differences between the Vitamin D Group and the Standard Treatment Group

#### 3.3.1 Comparison of Serological Difference Values between the Two Groups *after Adjustment for Confounders*

To control for potential confounding factors, multivariable linear regression analyses were performed, adjusting for age, sex, hypertension, diabetes, and cardiovascular and cerebrovascular diseases. The results indicated that, compared to the standard treatment group, the vitamin D group exhibited significantly greater improvements in immune and inflammatory profiles (Table **3**). Specifically, the vitamin D group showed a more pronounced increase in LYM levels (β = 0.32, *P* = 0.021) and significantly greater reductions in IL-6 (β = 5.62, *P* = 0.003) and CRP (β = 7.23, *P* = 0.007). Notably, after adjusting for comorbidities, the change in NEUT levels also showed a significant difference between the two groups (*P* = 0.026), a finding that was not observed in the unadjusted analysis (data not shown). Furthermore, the correction of vitamin D status was confirmed, with the vitamin D group showing significantly greater increases in 25(OH)D and serum calcium levels (*P* < 0.001). No significant differences were observed in the changes in WBC and PCT between the groups (*P* > 0.05).

#### 3.3.2 Comparison of Symptom Resolution Time and Viral Clearance Time between the Two Groups

**Fig. (1):**
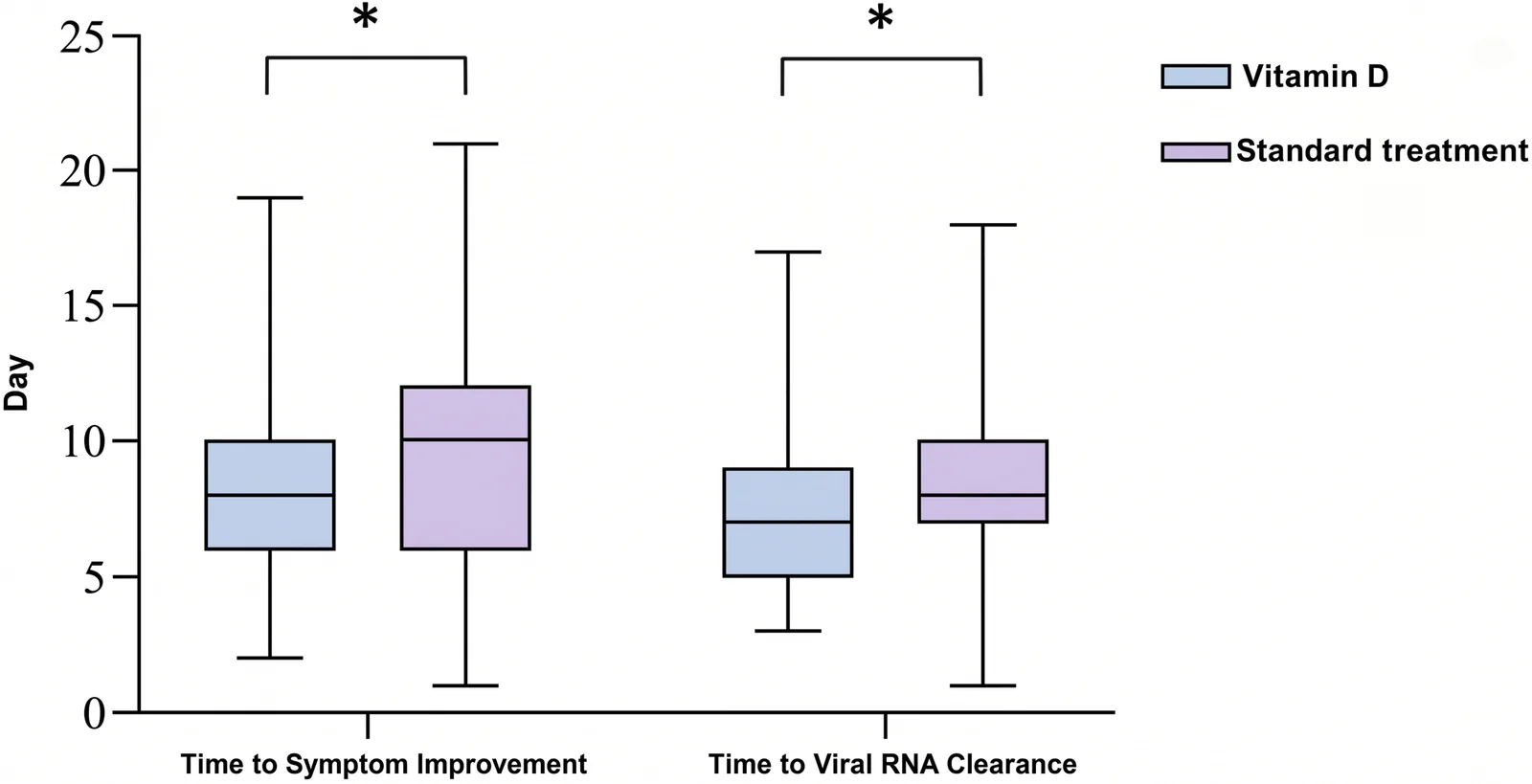
Comparison of time to symptom resolution and time to average viral clearance between the two groups. After treatment, the time to symptom resolution and time to average viral clearance in COVID-19 patients in the vitamin D group were shorter than those in the Standard Treatment Group. * *P* < 0.05 in inter-group comparison.

The average symptom resolution time for the vitamin D group was 7.00 days, and the average viral clearance time was 7.00 days. The average symptom resolution time for the standard treatment group was 10.00 days, and the average viral clearance was 8.00 days. The average symptom resolution time and average viral clearance time in the vitamin D group were both shorter than in the standard treatment group (*P* < 0.05) (Fig. **1**).

#### 3.3.3 Comparison of Imaging Changes between the Two Groups

Improvement was considered effective treatment, while no change and worsening were considered ineffective treatment. Compared to the standard treatment group, the vitamin D group showed more significant imaging improvement (*P* < 0.05) (Table **4**).

## 4 DISCUSSION

Coronaviruses are enveloped, positive-sense, single-stranded RNA viruses of approximately 30 kb. After infecting animals, coronaviruses can mutate and adapt, leading to the co-evolution of coronaviruses and the emergence of new human coronaviruses [23, 24], such as SARS-CoV-2, which caused the novel coronavirus pneumonia in 2019. Studies show that the plasma concentrations of cytokines and chemokines IL-1B, IL-1RA, IL-7, IL-8, IL-9, IL-10, basic fibroblast growth factor (bFGF), granulocyte colony-stimulating factor (GCSF), granulocyte-macrophage colony-stimulating factor (GMCSF), interferon (IFN)γ, and 10 kDa in COVID-19 patients are all higher than in healthy adults [25, 26]. Furthermore, plasma concentrations of interferon-inducible protein (IP)-10, GCSF, monocyte chemotactic protein (MCP)-1, active macrophage inflammatory protein 1 alpha (MIP1a), tumor necrosis factor α (TNFα), IL-2, IL-7, and IL-10 are higher in COVID-19 patients who require intensive care unit admission than in non-severe patients [27-29], indicating that the pathogenesis of COVID-19 is related to the body’s inflammatory response, and that the cytokine storm is linked to disease severity. The study by Wu *et al*. [30] further analyzed the LYM profile and found that the counts of CD3^+^ T LYM, CD3^+^CD4^+^ T LYM, CD3^+^CD8^+^ T LYM, CD19^+^ B LYM, and CD16^+^CD56^+^ NK cells in the peripheral blood of COVID-19 patients were significantly reduced. Except for CD16^+^CD56^+^ NK cells, the percentages of these LYM subsets increased, suggesting that peripheral blood T LYM, B LYM, and NK cells are all reduced in COVID-19 patients, with a more significant reduction in NK cells [31, 32]. Moreover, compared to patients with mild COVID-19, patients with severe COVID-19 had lower peripheral counts of CD3^+^ T LYM, CD3^+^CD4^+^ T LYM, CD3^+^CD8^+^ T LYM, and CD16^+^CD56^+^ NK cells, indicating that lymphopenia is a common feature in COVID-19 patients and may be a key factor associated with disease severity and mortality. The above research evidence indicates that the pathogenic mechanism of COVID-19 is related to immune dysregulation and an inflammatory cytokine storm caused by the excessive release of inflammatory factors.

Several retrospective studies have confirmed, from different perspectives, a link between vitamin D levels and the risk of COVID-19 infection. A study by Avolio *et al*. [33] found that 25(OH)D levels were significantly lower in COVID-19 patients than in the negative control group. Another study showed that patients with vitamin D deficiency had a higher risk of COVID-19 infection compared to those with sufficient vitamin D [34]. Furthermore, a multivariate analysis by Merzon *et al*. [10] showed an association between low 25(OH)D levels and an increased risk of COVID-19 infection. In a univariate analysis, low 25(OH)D levels were associated with an increased hospitalization rate for COVID-19, suggesting that low 25(OH)D levels are an independent risk factor for COVID-19 infection and hospitalization [35]. Additionally, vitamin D receptor (VDR) gene polymorphisms are related to COVID-19 susceptibility and poor outcomes [36, 37]. A case-control study showed that the VDR Fokl polymorphism might affect an individual's susceptibility to COVID-19 [38]. The study by Apaydin *et al*. [38] found that the Ff subtype of FokI (rs2228570) polymorphism was more common in moderate-to-severe COVID-19 patients, while the FF subtype was more common in patients with a better prognosis. The TT subtype of TaqI (rs731236) polymorphism was associated with poor outcomes requiring intensive care unit admission, while the Tt subtype could reduce the risk of ICU admission. The aa subtype of ApaI (rs7975232) polymorphism was more common in deceased patients and was associated with an increased COVID-19 mortality rate.

Some literature reports indicate that WBC, neutrophil percentage, NEUT, CRP, PCT, and IL-6 are elevated in COVID-19 patients, while RBC, hemoglobin, LYM percentage, eosinophil percentage, LYM count, eosinophil count, and platelet count are decreased [22, 39-41]. The results of this study show that the overall LYM counts in both groups were low before treatment, and the overall levels of WBC, NEUT, and PCT were not significantly elevated. This is likely because it was an early stage of a viral infection, not yet complicated by a bacterial infection. CRP is an indicator of the body's inflammatory response after pathogen infection, suggesting a strong inflammatory response in COVID-19 patients. IL-6 may play a key role in triggering the cytokine storm in COVID-19 patients [42]. In this study, the overall levels of IL-6 and CRP in both groups were higher than the normal reference upper limit. Furthermore, this study found that the overall 25(OH)D levels in both groups were below the normal lower limit, further confirming a close relationship between vitamin D levels and COVID-19, with inflammation likely playing an important role in between.

A cohort study by Oristrell *et al*. [16] showed that compared to 25(OH)D-deficient patients who did not receive vitamin D supplementation, those who received cholecalciferol treatment to raise their 25(OH)D levels to ≥30 ng/ml had a lower risk of SARS-CoV2 infection, a lower risk of severe COVID-19, and a lower mortality rate. Additionally, compared to COVID-19 patients receiving daily oral cholecalciferol, those who received a bolus of cholecalciferol had a lower mortality rate. A quasi-experimental study in a French nursing home showed that vitamin D supplementation in the elderly during COVID-19 illness was associated with less severe disease and higher survival rates [22], suggesting that vitamin D supplementation can provide a potential benefit for COVID-19 patients. This study used vitamin D2 injection to increase serum vitamin D concentration in COVID-19 patients. As vitamin D2 does not significantly affect 25(OH)D measurements in the short term, we monitored patients' serum calcium levels to assess the short-term effects of vitamin D supplementation. The results showed that serum calcium levels in the vitamin D group improved significantly compared to before treatment, while there was no significant change in serum calcium levels in the standard treatment group. A re-examination of 25(OH)D levels one month later showed a significant improvement in the vitamin D group compared to before treatment, while the standard treatment group also showed a slight increase in 25(OH)D levels. A further comparison of 25(OH)D improvement between the two groups found that the increase in 25(OH)D levels was more significant in the vitamin D group, indicating that vitamin D supplementation was effective. The slight increase in 25(OH)D levels in the standard treatment group may be due to factors such as dietary supplementation and sun exposure outside the hospital. Based on this, this study compared the improvement of various indicators in the two groups before and after treatment. The results showed that blood routine and infection indicators improved significantly in both groups. A further comparison of the changes in each indicator between the two groups found no significant difference in the improvement of WBC and PCT levels, but the changes in NEUT, LYM, IL-6, and CRP levels were more significant in the vitamin D group. Symptom duration and time required for viral clearance were shorter, and imaging outcomes were more likely to show improvement, indicating that vitamin D supplementation can improve inflammation in COVID-19 patients and has anti-inflammatory and antiviral effects.

Vitamin D plays a crucial role in regulating immune responses and can influence the occurrence and progression of COVID-19 by modulating both innate and adaptive immunity [43, 44]. 1,25(OH)2D binds to the VDR and vitamin D response elements to maintain barrier integrity by inducing the expression of proteins that maintain cell junctions [45-47], protecting the body from pathogen invasion. A study by Wang *et al*. [48] found that vitamin D response elements are present in the promoters of the cathelicidin antimicrobial peptide (CAMP) and defensin β2 genes, and vitamin D can induce CAMP gene expression in isolated human keratinocytes, monocytes, and neutrophils, as well as in human cell lines, thereby promoting the production of CAMP and defB2 to exert its antibacterial, antiviral, and immunomodulatory effects. Another property of vitamin D related to antimicrobial and antiviral mechanisms is its ability to promote autophagy [49, 50]. Autophagy is a fundamental biological process in which damaged organelles, misfolded proteins, and viral particles are enclosed by intracellular membranes, which then fuse with lysosomes to form autophagolysosomes, degrading the enclosed contents to clear pathogens and maintain cellular homeostasis [51-54]. Both 25(OH)D and 1,25(OH)2D binding to VDR can enhance the expression of the autophagy marker protein LC3 [55, 56]. Beyond VDR-mediated signaling and autophagy induction, vitamin D has been shown to modulate several COVID-19-relevant pathways. For instance, 1,25-dihydroxyvitamin D enhances the expression of the antimicrobial peptide cathelicidin (LL-37), which not only exhibits direct antiviral activity but also binds to the SARS-CoV-2 spike protein and inhibits its attachment to the ACE2 receptor—a critical step in viral entry (Vitamin D-regulated gene expression profiles: Species-specificity and cell-specific effects on metabolism and immunity). Moreover, vitamin D helps regulate the renin-angiotensin system (RAS) by upregulating ACE2 expression, thereby potentially attenuating acute lung injury and cytokine-driven pathology (Vitamin D alleviates lipopolysaccharide-induced acute lung injury via regulation of the renin-angiotensin system). Vitamin D also suppresses pro-inflammatory cytokines such as IL-6 and TNF-α via inhibition of NF-κB and MAPK pathways, and promotes a shift from pro-inflammatory Th1/Th17 responses toward immunomodulatory Treg differentiation (Mechanisms in endocrinology: vitamin D and COVID-19; Correlation between premorbid IL-6 levels and COVID-19 mortality: Potential role for Vitamin D. International Immunopharmacology; Cathelicidins Modulate TLR-Activation and Inflammation). These mechanisms collectively contribute to mitigating the cytokine storm and immune exhaustion commonly observed in severe COVID-19.

1,25(OH)2D can inhibit the expression of major histocompatibility complex (MHC) II molecules [57] and co-stimulatory molecules 40, 80, and 86, and also inhibit the maturation, differentiation, survival, and antigen-presenting function of dendritic cells (DC), leading to reduced antigen presentation and T cell activation, and subsequently, inhibition of adaptive immune responses [57-60]. 1,25(OH)2D also regulates DC-derived cytokine expression by inhibiting the release of IL-12 and IL-23 [61]. Furthermore, 1,25(OH)2D can directly affect the function of different T cell subsets, causing the body to switch from a pro-inflammatory immune state to an immune-tolerant state [62, 63]. 1,25(OH)2D can inhibit the proliferation of Th1 and Th17 cells and the abnormal release of pro-inflammatory cytokines [64-66], mediate the differentiation of Th2 cells and the release of their anti-inflammatory cytokines, and promote Treg differentiation by directly targeting T cells via DC regulation, leading to a shift from a Th1/Th17 phenotype to a Th2/Treg cell phenotype [67], which prevents over-activation of the immune response that can cause immune imbalance.

Additionally, vitamin D can upregulate the expression of NF-κB signaling pathway inhibitory proteins [68], leading to a decrease in IFN-β production and downregulation of interferon-stimulated gene mRNA expression [49, 69], thereby inhibiting the inflammatory response. Mitogen-activated protein kinase phosphatase (MKP) can dephosphorylate the threonine and tyrosine residues of activated mitogen-activated protein kinase (MAPK), leading to MAPK inactivation [70, 71]. The study by Zhang *et al*. [72] found that vitamin D inhibits the MAPKs P38 signaling pathway by upregulating MKP-1 expression, thereby reducing the production of pro-inflammatory cytokines. IL-6 is associated with severe poor outcomes in patients with SARS-CoV-2 pneumonia and plays a key role in the COVID-19 cytokine storm [73-75]. The above studies indicate that vitamin D can improve the inflammatory response in COVID-19 patients by reducing the release of pro-inflammatory factors like IL-6, which is consistent with the results of this study.

Since its outbreak in 2019, COVID-19 has quickly swept the globe, affecting the health of people of all ages. To this day, COVID-19 still has small-scale outbreaks during the autumn and winter, making the prevention of COVID-19 infection and the reduction of its severity a hot public issue. Vitamin D deficiency is linked to COVID-19 susceptibility and disease severity. International studies show that vitamin D supplementation can reduce COVID-19 susceptibility and improve poor outcomes, but due to significant differences in the included populations, vitamin D supplementation methods, and dosages, their results have not reached a consensus. Currently, there are no published studies in China on the use of vitamin D supplementation for COVID-19 treatment. This study collected pre- and post-treatment infection indicators from Han Chinese COVID-19 patients and compared the improvement of these indicators after vitamin D supplementation, with the hope of providing some reference for the treatment and prevention of COVID-19.

This study has some limitations: 1) The included population consisted of patients with co-morbid chronic diseases such as diabetes, hypertension, and coronary heart disease, which may constitute confounding factors; 2) The study subjects were limited to mild COVID-19 patients with vitamin D insufficiency/deficiency and did not include severe/critical patients or those with sufficient vitamin D; 3) We only evaluated the short-term efficacy of a single intramuscular injection of vitamin D2 (15 mg) and did not compare different supplementation methods, dosages, or assess long-term effects; 4) This study was a retrospective observational study conducted during a specific period, and further research is needed to investigate the long-term effects of vitamin D supplementation on COVID-19 patients.

Fig. (**1**) Comparison of Time to Symptom Resolution and Time to Average Viral Clearance Between the Two Groups After treatment, the time to symptom resolution and time to average viral clearance in COVID-19 patients in the Vitamin D group were shorter than those in the Standard Treatment Group. * *P* < 0.05 in inter-group comparison.

## CONCLUSION

This study provides evidence that adjunctive vitamin D supplementation confers significant benefits to COVID-19 patients beyond standard care. It not only accelerates virological clearance and symptom resolution but also effectively modulates the inflammatory response and mitigates lung injury, as evidenced by improved serological and imaging outcomes. Therefore, correcting vitamin D deficiency may represent a straightforward and valuable strategy in the comprehensive management of COVID-19.

## Figures and Tables

**Table 1:** Comparison of pre-treatment baseline characteristics between the standard treatment group and the vitamin D group.

-	**Standard Treatment Group**	**Vitamin D Group**	***P***
Gender (Male/Female)	89/61	85/65	0.640
Age (Years)	51.50 (40.00, 65.00)	55.00 (43.75, 66.25)	0.296
WBC (×10^9^/L)	7.75 ± 2.03	8.22 ± 3.02	0.119
NEUT (×10^9^/L)	6.76 ± 2.16	7.31 ± 3.40	0.099
LYM (×10^9^/L)	1.23 ± 0.41	1.18 ± 0.49	0.369
IL-6 (pg/mL)	25.39 ± 6.79	27.52 ± 13.87	0.092
CRP (mg/L)	51.18 ± 9.39	54.25 ± 20.05	0.090
PCT (ng/mL)	0.68 ± 0.31	0.72 ± 0.36	0.254
25 (OH)D (ng/mL)	12.89 ± 3.05	13.81 ± 5.33	0.066
Serum Calcium (mmol/L)	2.17 ± 0.08	2.15 ± 0.15	0.065

** Abbreviation:** WBC, white blood cell; NEUT, neutrophil; LYM, lymphocyte; IL-6, interleukin-6; CRP, c-reactive protein; PCT, procalcitonin.

**Table 2:** Comparison of parameters before and after treatment in the vitamin D group and the standard treatment group.

-	**Standard Treatment Group**		**Vitamin D Group**	
	Pre-treatment	Post-treatment	Pre-treatment	Post-treatment
WBC (×10^9^/L)	7.75 ± 2.03	7.16 ± 2.07*	8.22 ± 3.02	7.50 ± 2.07*
NEUT (×10^9^/L)	6.76 ± 2.16	6.06 ± 3.32*	7.31 ± 3.40	6.43 ± 3.42*
LYM (×10^9^/L)	1.23 ± 0.41	1.39 ± 0.48*	1.18 ± 0.49	1.51 ± 0.61*
IL-6 (pg/mL)	25.39 ± 6.79	7.97 ± 5.60*	27.52 ± 13.87	7.42 ± 6.62*
CRP (mg/L)	51.18 ± 9.39	18.48 ± 6.55*	54.25 ± 20.05	16.40 ± 6.66*
PCT (ng/mL)	0.68 ± 0.31	0.24 ± 0.17*	0.72 ± 0.36	0.25 ± 0.17*
25 (OH) D (ng/mL)	12.89 ± 3.05	13.16 ± 3.03*	13.81 ± 5.33	16.70 ± 4.86*
Serum Calcium (mmol/L)	2.18 ± 0.08	2.17 ± 0.07	2.15 ± 0.15	2.19 ± 0.14*

**Note:** * P < 0.05 compared to pre-treatment within the same group.

**Table 3:** Multivariable linear regression analysis of changes in serological parameters and vitamin D status between the vitamin D group and the standard treatment group.

-	** Standard Treatment Group **	** Vitamin D Group **	** Adjusted β ** ** (95% CI) ^†^**	***P***
WBC (×10^9^/L)	0.59 ± 2.97	0.72 ± 3.00	-0.22 (-1.22, 0.14)	0.528
NEUT (×10^9^/L)	0.70 ± 4.22	0.87 ± 3.87	-0.89 (-1.67, -0.11)	0.026*
LYM (×10^9^/L)	-0.16 ± 0.61	-0.33 ± 0.59	0.32 (0.05, 0.59)	0.021*
IL-6 (pg/mL)	17.42 ± 8.43	20.11 ± 11.70	-5.62 (-9.34, -1.90)	0.003*
CRP (mg/L)	32.70 ± 10.54	37.86 ± 18.28	-7.23 (-12.45, -2.01)	0.007*
PCT (ng/mL)	0.43 ± 0.38	0.48 ± 0.28	0.04 (-0.04, 0.11)	0.343
25 (OH)D (ng/mL)	-0.27 ± 0.74	-2.89 ± 1.07	-2.71 (-2.90, -2.52)	<0.001*
Serum Calcium (mmol/L)	0.01 ± 0.07	-0.04 ± 0.15	-0.056 (-0.083, -0.030)	<0.001*

**Note:** ^†^ The adjusted β coefficients and *P* values were derived from multivariable linear regression models, with the Standard Treatment Group as the reference, adjusting for age, sex, hypertension, diabetes, cardiovascular and cerebrovascular diseases.* *P* < 0.05.

**Table 4:** Comparison of imaging improvement between the vitamin D group and the standard treatment group.

-	**Number of Cases (n)**	**Improvement (%)**	**No Change (%)**	**Worsening (%)**	**Effective (%)**
Standard Treatment Group	150	15 (10.0)	115 (76.7)	20 (15.0)	15 (10.0)
Vitamin D Group	150	73 (48.7)	67 (44.7)	10 (6.7)	73 (48.7)
c^2^	-	-	-	-	54.22
*P*	-	-	-	-	<0.001*

**Note:** *P*<0.001*.

## Data Availability

Not applicable.

## LIST OF ABBREVIATIONS

COVID-19: Coronavirus Disease 2019
ARDS: Acute Respiratory Distress Syndrome
WBC: White Blood Cell
NEUT: Neutrophil
LYM: Lymphocyte
IL: Interleukin
CRP: C-Reactive Protein
PCT: Procalcitonin
bFGF: Basic Fibroblast Growth Factor
GCSF: Granulocyte Colony-Stimulating Factor
GMCSF: Granulocyte-Macrophage Colony-Stimulating Factor
IFN: Interferon
IP: Interferon-Inducible Protein
MCP: Monocyte Chemotactic Protein
MIP-1α: Macrophage Inflammatory Protein-1 Alpha
TNF-α: Tumor Necrosis Factor Alpha
VDR: Vitamin D Receptor
MHC: Major Histocompatibility Complex
DC: Dendritic Cells
CAMP: Cathelicidin Antimicrobial Peptide
MKP: Mitogen-Activated Protein Kinase Phosphatase
MAPK: Mitogen-Activated Protein Kinase

## STANDARDS OF REPORTING

STROBE guidelines were followed.
